# Estimation of *PEX1*-mediated Zellweger spectrum disorder births and population prevalence by population genetics modeling

**DOI:** 10.1016/j.gimo.2025.103431

**Published:** 2025-04-23

**Authors:** Karen E. Malone, Catherine Argyriou, Evelyn Zavacky, Nancy Braverman

**Affiliations:** 1 GeneScape, Leiden, The Netherlands; 2 Department of Human Genetics, McGill University, Montreal, QC, Canada; 3 Research Institute of the McGill University Health Center, McGill University, Montreal, QC, Canada; 4 Department of Human Genetics and Department of Pediatrics, McGill University, Montreal, QC, Canada

**Keywords:** Heimler syndrome, Peroxisome biogenesis factor 1, PEX1, Zellweger spectrum disorder

## Abstract

**Purpose:**

Zellweger Spectrum Disorder (ZSD) is a rare syndromic disorder characterized by impaired peroxisome assembly and function. Many cases are due to pathogenic variants in the *PEX1* gene and are inherited in an autosomal recessive manner. As with many rare diseases, understanding the disease burden and scale of unmet need is challenging but required to support diagnosis, disease management, and development of therapies. We present a population-genetics-based model to estimate births and overall disease prevalence for patients in the United States, European countries, and Japan.

**Methods:**

We utilized large-scale genetic diversity data sets to estimate the mutational burden per region and integrated genotype-phenotype relationships with real-world survival data to provide patient number estimates for severe, intermediate, and mild segments per age and country.

**Results:**

We observed regional differences in the variant landscapes expected to contribute to *PEX1*-mediated ZSD (*PEX1*-ZSD). Conservative prevalence estimates for the United States, United Kingdom, Germany, France, Italy, Spain, and Japan based solely on known pathogenic variants indicates nearly 500 patients in total. Incorporating predicted pathogenic variants into our model suggests an additional 260 patients with intermediate phenotype and 930 patients with mild phenotype, under the age of 30, across these countries.

**Conclusion:**

Notably, our model indicates that a significant proportion of patients with intermediate/mild phenotype may go unrecognized by current diagnostic practices. This diagnosis independent model of patient number estimates provides additional insights into the broad spectrum of *PEX1*-ZSD on a more global scale and can be used to inform health care strategies for these patients.

## Graphical abstract

**Figure:**
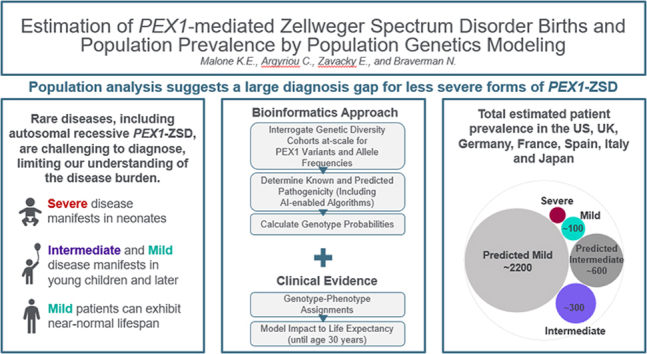

## Introduction

Zellweger spectrum disorder (ZSD) is a heterogeneous multisystemic disorder caused by deleterious variants in any of 13 *PEX* genes, whose protein products are required for peroxisome assembly and function.^1^ Inheritance is primarily autosomal recessive, although additional modes have recently been described for specific heterozygous variants in *PEX6* (HGNC:8859) and *PEX14* (HGNC:8856).^2^^,^^3^ PEX protein deficiency affects multiple downstream metabolic pathways, primarily the metabolism of complex lipids, and results in a variable clinical presentation that can include brain, eye, cochlear, liver, adrenal, renal, and bone involvement.^1^^,^^4^ Around 30% of ZSD patients have *PEX* gene null variants resulting in nonfunctional PEX protein and congenital brain malformations and do not survive beyond infancy. In contrast, the majority (70%) have an intermediate-milder phenotype due to residual PEX protein function that is characterized by peroxisome dysfunction over postnatal life and its chronic effect on tissue and organ homeostasis.^1^

The majority of ZSD (estimated 70%) is caused by deleterious variants in the *PEX1* gene (HGNC:8850),^5^ which encodes a AAA-ATPase (ATPases associated with various cellular activities) required to maintain peroxisome enzyme import.^6^ Patients with milder forms of *PEX1*-mediated ZSD (*PEX1*-ZSD) can have normal intellect or mild cognitive deficiency, survive to adulthood, can be employed, and can live semi-independently or independently.^7^, ^8^, ^9^ In contrast, patients with intermediate severity have moderate cognitive delays, most develop adrenal insufficiency, and around 25% develop additional disabilities, including epilepsy and leukodystrophy.^1^ Survival depends largely on the neurologic involvement, but many survive into adulthood.^10^ Regardless of severity, all patients with *PEX1*-ZSD develop sensorineural hearing loss and progressive retinopathy leading to blindness^11^^,^^12^ (OMIM 234580, 214100, and 601539).

Despite increased diagnosis and improved understanding of the underlying pathology driving ZSD, articulating the scale of unmet need for this rare and variably presenting disorder remains challenging, as with many rare disease indications. Nevertheless, reliable prevalence and geographic estimates for target patients are integral for the development and commercialization of innovative therapies for such indications. Multiple barriers to diagnosis and labeling in rare diseases further hamper disease estimations by conventional methods and real-world evidence approaches. However, because *PEX1*- ZSD is an autosomal recessive disorder, it is possible to estimate prevalence of pathogenic genotypes based on allele frequencies from heterozygous persons, provided that sufficiently large, representative cohorts are available. Critically, this approach is independent of diagnosis and provides an indication of the diagnosis gap that is anticipated in most rare diseases, including ZSD.

Based on the current knowledge concerning genotype-phenotype relationships in *PEX1*- ZSD, their impact on life expectancy, and the availability of genetic diversity data, we have modeled the birth prevalence and overall disease prevalence for the United States, United Kingdom, Germany, France, Italy, Spain, and Japan. This model paints the most up-to-date picture of the scale of unmet need for *PEX1*-ZSD across multiple geographies and phenotypes.

## Materials and Methods

### Allele frequencies for variants in the PEX1 gene from representative cohorts

**Supplemental Figure 1:**
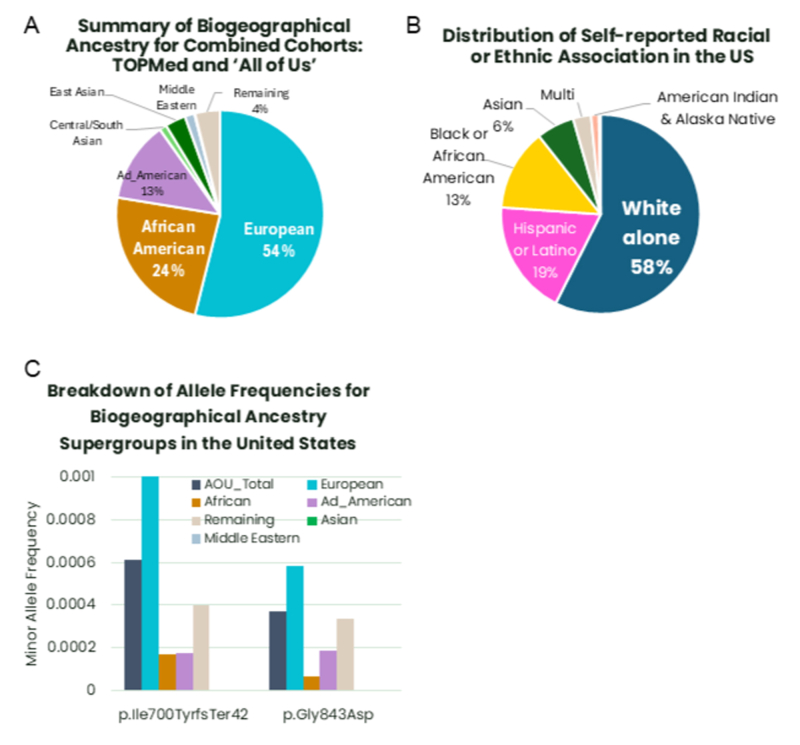


Minor allele frequencies for variants in the *PEX1* gene were obtained from multiple sources that capture the genetic diversity of the target regions. To represent the genetic diversity of the United States, we combined TOPMed (data freeze 10)^13^ with the recent All of Us^14^ genetic diversity data set (Supplemental Figure 1). To capture the genetic diversity of United Kingdom, we utilized the UK Biobank (Li S, Carss KJ, Halldorsson BV, Cortes A, Consortium U. Whole-genome sequencing of half-a-million UK Biobank participants. medRxiv. Published online January 1, 2023:2023.12.06.23299426. https://doi.org/10.1101/2023.12.06.23299426). For European countries, we utilized the non-Finnish European cohort of gnomAD v4, excluding the UK Biobank, and further informed country models with data from gnomad v2 and v3,^15^ Northwestern European, and Southern European subcohorts. Japan is represented by the JPN_UNI aggregated cohorts obtained from TogoVAR.^16^ Density plots of allele frequencies across European geography for all available cohorts were done using Surfer package (version 25.1.229 from Golden Software LLC) for the 2 primary variants; NC_000007.14:g.92503172dup, NM_000466.3:c.2097dup, p.(Ile700fs∗42) and NC_000007.14:g.92501562C>T, NM_000466.3:c.2528G>A, p.(Gly843Asp), see Supplemental Table 1).

### Inclusion of PEX1 variants in the models and prediction of pathogenic variants

Variants of known pathogenicity were identified through cross-reference with ClinVar, as designated by pathogenic/likely pathogenic tags, and conversely, known benign variants were excluded. These confirmed pathogenic variants were the basis of our core patient population models and are listed in Supplemental Table 2.

In addition, given the heterogeneity of *PEX1*-ZSD, we also anticipate more variants to contribute to the total patient population than have been recognized in the clinical setting. To capture putative pathogenic variants, we evaluated all *PEX1* variants identified in the large cohorts by multiple in-silico-based algorithms using Ensembl VEP^17^ as herein described. Specifically, high-confidence predicted loss-of-function variants (pLOF) were identified as premature stop variants, frameshifts, start lost, and essential splice donor/acceptor variants (American College of Medical Genetics and Genomics [ACMG] evidence: PVS1 and PM2). These high-confidence pLOF variants were also applied to the core patient population models and listed in Supplemental Table 3.

Further potentially pathogenic variants that may retain some residual PEX1 function were also predicted as described below, and these nonnull variants were applied to our expanded patient population models. Potential cryptic splice variants (Supplemental Table 4) were identified with SpliceAI^18^ applying the high precision cutoff of maximum delta scores of 0.8 or higher and interpretation for impact to splicing using the SpliceAI-10K calculator^19^ (ACMG Evidence: PM2 and PP3). To incorporate potentially pathogenic missense variants, we applied consensus voting to 4 algorithms; SIFT, Polyphen-2, EVE,^20^ and AlphaMissense^21^ (ACMG evidence: PM2 and PP3), which are listed in Supplemental Table 5. Furthermore, all predicted pathogenic variants incorporated in the models had an allele frequency ≤ 0.002, corresponding to the threshold defined in our training set, separating known pathogenic and benign variants. In addition, there were no homozygous persons identified in the genetic diversity cohorts for the predicted pathogenic variants. All variants included in this modeling are listed in Supplemental Tables 2 to 5.

### Genotype- phenotype assignments and estimation of annual birth prevalence

Based on extensive work in characterizing *PEX1*-ZSD patients,^8^^,^^10^^,^^22^ we assigned the genotype- phenotype relationships as described in Supplemental Table 6. Namely, genotypes attributed to the most pervasive *PEX1* variants, c. 2097_2098insT (which translates to p.(Ile700fs∗42), or “null”) and c.2528G>A p.(Gly843Asp) were designated as: p.(Ile700fs∗42) homozygous → severe phenotype; p.(Gly843Asp) homozygous → mild phenotype and p.[Ile700fs∗42]; [Gly843Asp] compound heterozygous → intermediate phenotype. To determine the total birth prevalence and ratio of specific genotype classes, the genotype prevalence was calculated using the expanded Hardy-Weinberg principle. The 90% confidence range was determined with the modified Wald method.^23^ The corresponding genotype prevalence was applied to the number of historical births in 2021 for a specified country to determine the estimated number of children born per year with a given genotype. Country-specific birth data were obtained from offices for vital records.^24^, ^25^, ^26^, ^27^

### Impact on life expectancy and conversion of birth incidence to disease prevalence

Depending on the associated phenotype, patients with ZSD may exhibit premature mortality or may have near-normal life expectancy. Based on previous Kaplan-Meier analyses of survival from Bose et al^10^ (2022), we prepared survival models for severe, intermediate, and mild phenotypes, using linear regression of a meta-analysis of the reported 2 longitudinal studies. Patients with severe ZSD generally do not survive past 5 years of age, and based on a linear model, we applied an average annual survival of 76.0%. Intermediate phenotype patients also exhibit reduced survival in the first decade of life. However, those that live into the second decades and beyond exhibit relatively improved survival. This was modeled as annual survival of 96.6% until age 8, 99.2% annual survival from ages 9 to 18, and 98.0% survival from 19 years and further. Mild patients exhibit near-normal life expectancy, with an annual survival rate estimated at 99.4%. Survival was only modeled up to 31 years of age because of limited data beyond this point.

For rare genotypes that may present as mild or intermediate phenotypes (Supplemental Table 6) but for which there is insufficient information to make a reliable assignment, we assumed for the purposes of modeling that half would present with intermediate phenotypes and half would present with mild phenotypes and randomly assigned patient segments to either intermediate or mild phenotypes. Historical birth data from countries were utilized to estimate the number of births per year by genotype and expected phenotype. Based on genotype-phenotype assignments, people were aged to present according to the survival models described above.

## Results

### Geographic distribution of pathogenic and predicted pathogenic PEX1 variants

**Figure 1:**
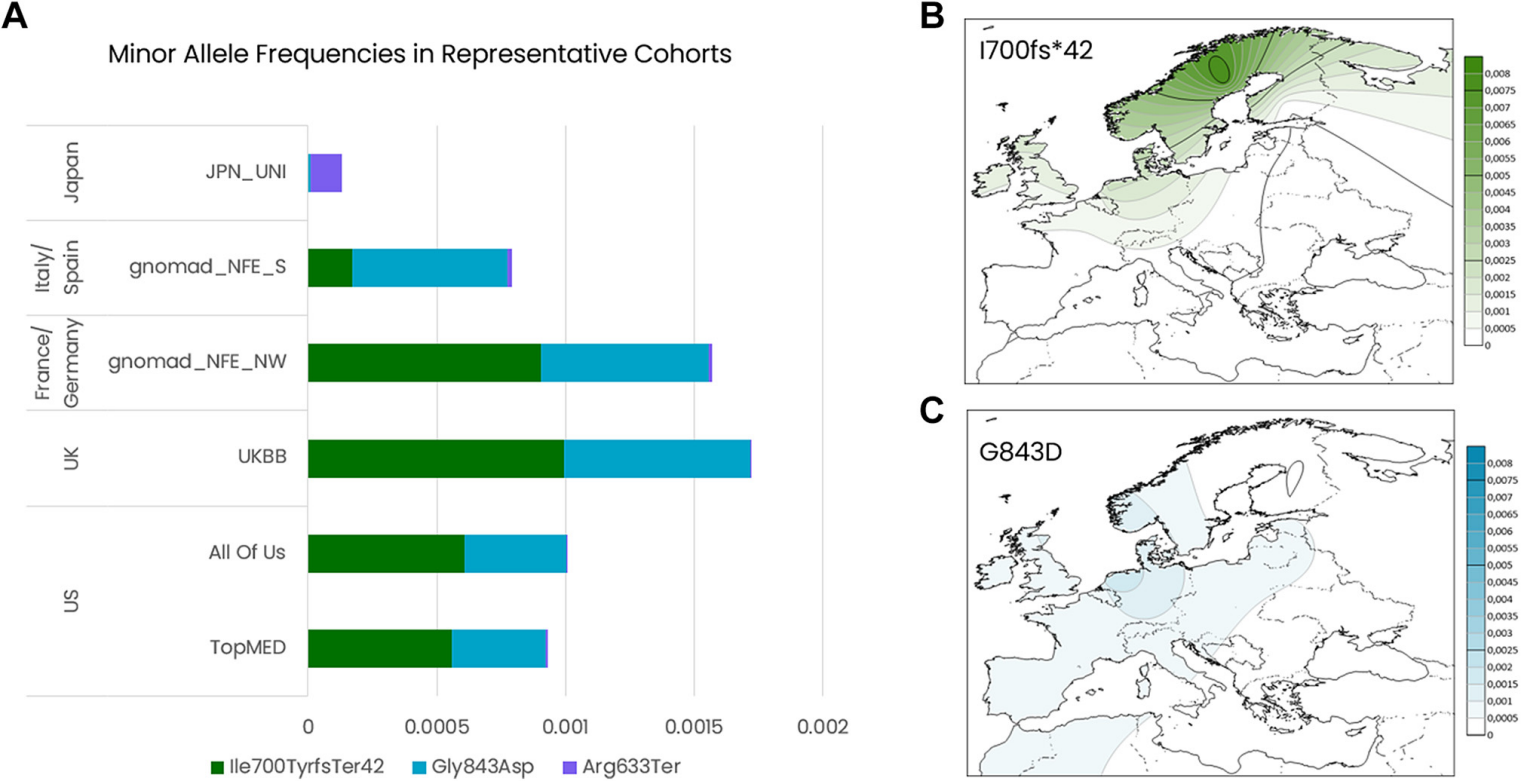
**Minor allele frequencies in representative cohorts.** A. The minor allele frequencies detected in the representative cohorts for the most pervasive pathogenic variants; JPN_UNI: aggregated data from TogoVAR; gnomad_NFE_S: non-Finnish European, Southern Europe subcohort; gnomad_NFE-NW: non-Finnish European, Northwestern Europe subcohort. Note. Variant p.(Arg633Ter) is also detected in western cohorts at very low frequency but is not readily visualized in this graph. B. Contour map of the variant allele frequencies across Europe for the most pervasive variants.

The 2 most pervasive *PEX1* pathogenic variants known in western countries are p.(Ile700fs∗42) and p.(Gly843Asp). These variants and patients have been well characterized. Patients who are homozygous for either p.(Ile700fs∗42) or p.(Gly843Asp) exhibit contrasting phenotypes, with p.(Ile700fs∗42) homozygous patients associated with severe impairment and poor prognosis, whereas p.(Gly843Asp) homozygous patients exhibit milder phenotype.^10^^,^^22^^,^^28^ A large proportion of compound heterozygous patients for these 2 variants have also been reported and are described as an intermediate phenotype.^10^^,^^22^^,^^28^ We observed that p.(Gly843Asp) is relatively common throughout Europe and less so in the US cohorts, whereas p.(Ile700fs∗42) is enriched in Northwestern European genetic ancestries, including the United Kingdom, and found in the United States. (Figure 1A). The geographies of these 2 variants are further represented by the inferred allelic distribution mapped across Europe (Figure 1B and C). Geographic mapping suggests that p.(Ile700fs∗42) originates in Scandinavian genetic ancestry before spreading into Northern Europe, whereas p.(Gly843Asp) is present at similar frequency throughout continental Europe.

Notably, but unsurprisingly, neither of these 2 variants are detected in the available Japanese cohorts. Instead, we identified NC_000007.14:g.92506251G>A, NM_000466.3:c.1897C>T, p.(Arg633Ter) (ClinVar: 550841) as the most prominent known pathogenic *PEX1* variant in this region (Figure 1A). This nonsense variant is previously reported in patients from Japan^29^ and Saudi Arabia^30^ and is associated with severe phenotypes when found in homozygosity. We also identified p.(Arg633Ter) heterozygous persons in western cohorts at very low frequency (Figure 1A). The complete listing of known pathogenic variants detected in target cohorts can be found in Supplemental Table 2.

**Figure 2:**
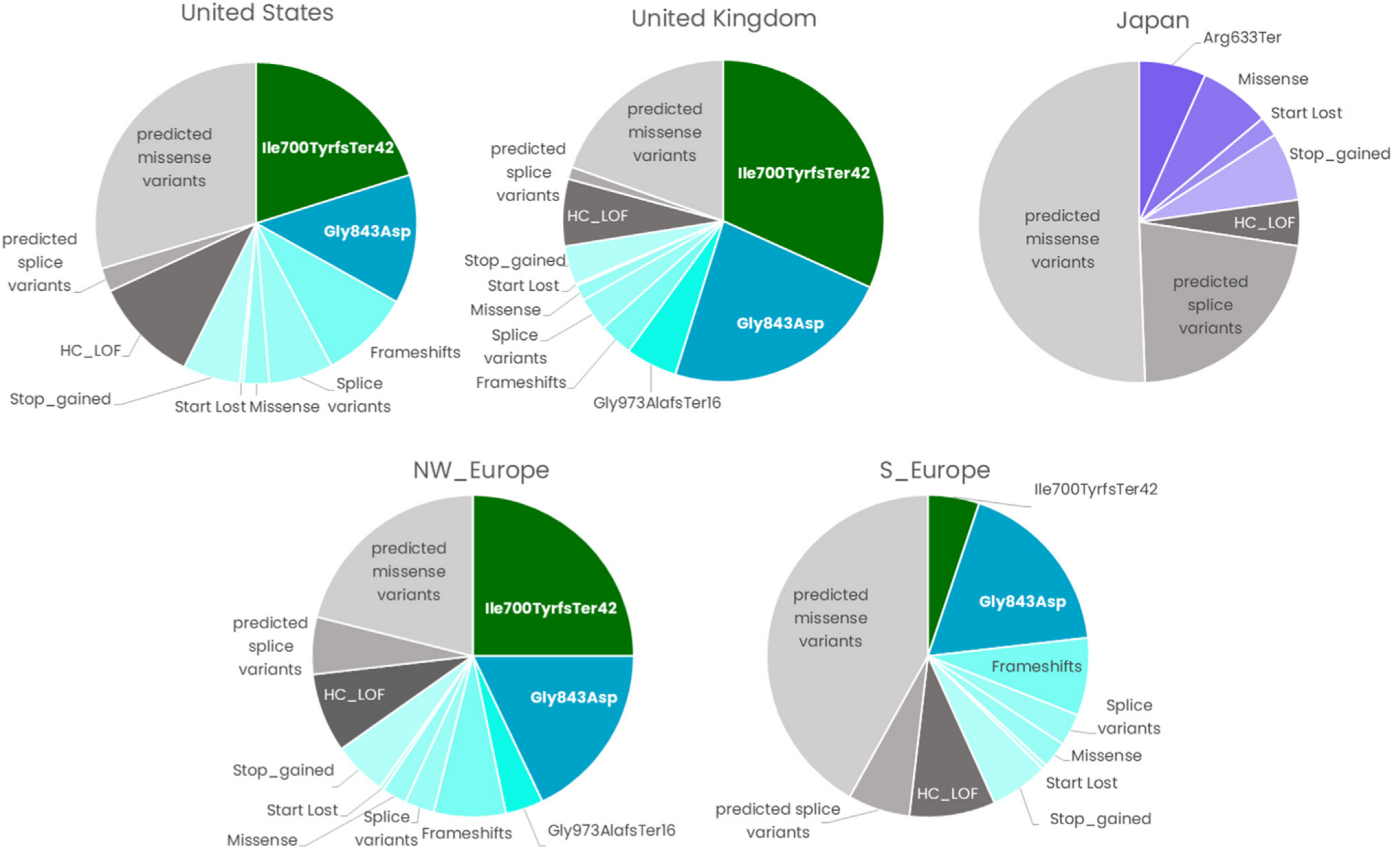
**Summary of the contribution of variants, both known and predicted pathogenic, applied to the population genetics-based models for *PEX1*-driven ZSD by region.** ZSD, Zellweger spectrum disorder.

In addition to known pathogenic variants as above, we also predicted variants that are expected to be pathogenic but have not necessarily been reported in the clinical setting. Figure 2 summarizes the contribution of known and predicted pathogenic variants in the primary models. It is clear that the United States, United Kingdom, and Northwestern Europe share significant genetic overlap, and more than half of the total expected mutational burden can be attributed to known pathogenic variants. In the complete overview, Japan exhibits a distinct mutational landscape from western regions. The full listings for predicted pathogenic variants that were included in the models and their presence in specific cohorts can be found in the Supplemental Tables 2 to 5.

### Annual birth prevalence

By applying Hardy-Weinberg principles and utilizing country-specific birth demographics, we calculated the total estimated number of children born per country in 2021 (Table 1). In our core models, we restricted our calculations to known pathogenic variants and high-confidence predicted loss-of-function variants (listed in Supplemental Tables 2 and 3); providing the most conservative estimates for these figures. The most children with *PEX1*-ZSD are expected to be born in the United States, United Kingdom, Germany, and France. When we also include predicted pathogenic missense variants and potential splice variants to calculate our expanded model (listed in Supplemental Tables 4 and 5), we expect considerably higher numbers of children in all countries that are modeled. The relatively higher prevalence ratios observed in northwestern European countries reflect the higher frequency of p.(Ile700fs∗42) and p.(Gly843Asp) in this geography (Table 2). Incorporation of predicted pathogenic missense variants and splice variants in our expanded models increased the estimated birth prevalence to similar rates across all regions, except for Japan. (Table 2).

**Table 1:** Estimated total number of *PEX1*-mediated ZSD births per country in 2021

Models	US	UK	Germany	France	Italy	Spain	Japan
Core model	15^a^ (13.8- 16.1)	4^a^ (4.0-4.6)	6^a^ (5.0-6.3)	5^a^ (4.7-5.8)	1^a^ (1.0-1.4)	1^a^ (0.9- 1.2)	<1^a^ (0.1- 0.3)
Expanded model	32^a^ (29.7-34.7)	7^a^ (6.4-7.2)	10^a^ (9.5-11.5)	10^a^ (8.9-10.7)	4^a^ (4.1-4.9)	4^a^ (3.4-4.2)	3^a^ (2.1-3.3)

Ninety percent confidence range in parentheses.

*UK*, United Kingdom; *US*, United States; *ZSD*, Zellweger Spectrum Disorder.

a Best whole number estimates.

**Table 2:** Estimated birth prevalence rate of *PEX1*-mediated ZSD per country

90% Confidence Range Per Million Births	US	UK	Germany	France	Italy	Spain	Japan
Core model	3.8-4.4	5.8-6.6	6.3-7.9	6.3-7.9	2.6-3.4	2.6-3.4	0.2-0.4
Expanded model	8.1-9.5	9.3-10.4	12.0-14.4	12.0-14.4	10.1-12.4	10.1-12.4	2.7-4.3

*ZSD*, Zellweger Spectrum Disorder.

### Genotype-phenotype assignments and conversion to population prevalence

**Figure 3:**
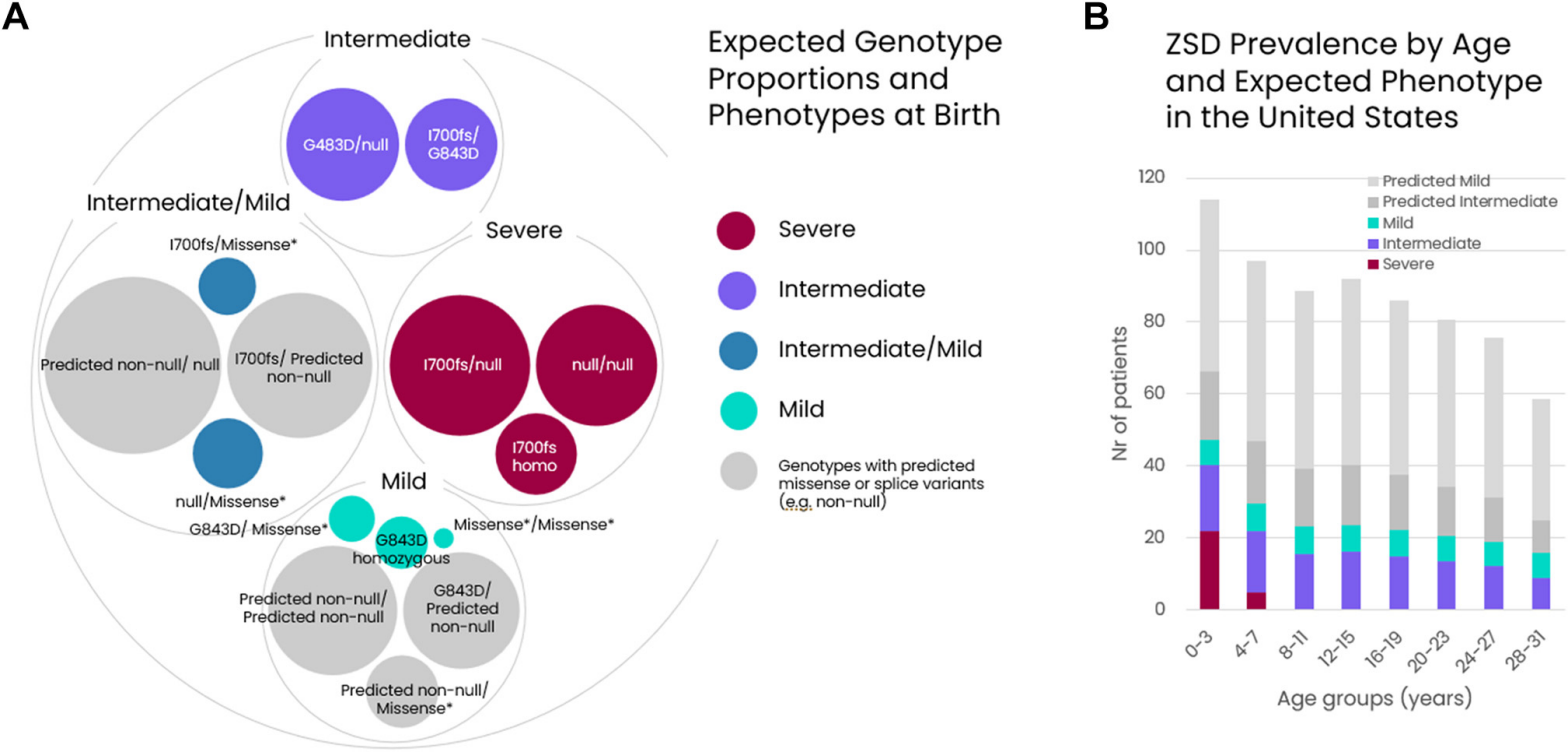
**Expected genotype proportions and phenotypes at birth and ZSDprevalence by age and expected phenotype in the United States.** A. Proportions of genotypes expected at birth in the United States and their corresponding expected phenotypes. Missense∗ denotes all other known pathogenic missense variants except G843D. B. Estimated current prevalence of all *PEX1*-driven ZSD patients in the United States stratified by age and expected phenotype. ZSD, Zellweger spectrum disorder.

Given the genetic and phenotypic heterogeneity of *PEX1*-ZSD, it is imperative to understand the genotype-phenotype relationships in this disease with respect to life expectancy to translate these data into the total population prevalence. As described in the methods, we assigned genotype-phenotype relationships based on the expected genotypes in each birth cohort. An example of our US model is shown in Figure 3A. We were able to designate 4 phenotypic buckets: severe, intermediate, mild, and combined intermediate/mild. The latter group is expected to be variant specific but there are insufficient examples to confidently assign specific phenotypes for rarely seen variants in our current model. Consequently, for conversion to population prevalence, we assumed that half of this group presented as intermediate and the other half as mild.

When we applied the specific genotype prevalence with phenotype assignments to the historical births per country and aged them to the present according to the observed survival rates, we were then able to estimate the current prevalence of *PEX1*-ZSD per country and expected phenotype, as well as their age distribution. The complete prevalence model for the United States is shown in Figure 3B. The total prevalence of *PEX1*-ZSD patients in the United States based on the core model of known pathogenic variants is estimated to be ∼200 patients under the age of 31. The majority of these are expected to exhibit an intermediate phenotype because severe patients have poor survival. When we consider the impact of predicted pathogenic variants, the estimated prevalence is substantially higher, with potentially an additional ∼120 predicted intermediate patients under the age of 31 and an additional ∼372 predicted mild patients in the United States.

**Figure 4:**
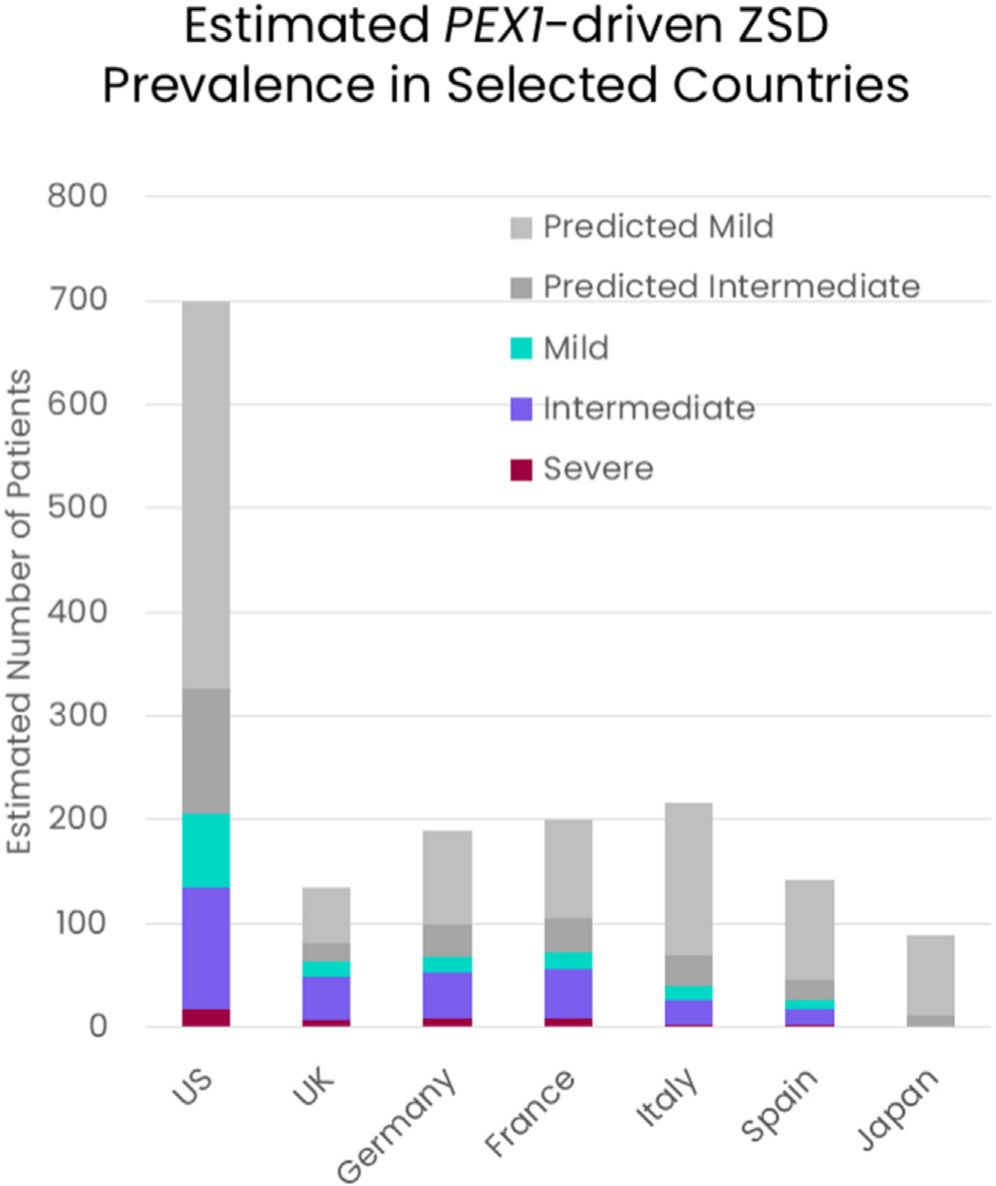
**Summary of the estimated current total prevalence of all *PEX1*-ZSD patients in the target countries, stratified by expected phenotype.** ZSD, Zellweger spectrum disorder.

Following the same approach, we also estimated the current disease prevalence for the United Kingdom, Germany, France, Italy, Spain, and Japan (Figure 4). The United Kingdom, Germany and France are anticipated to have some of the highest prevalence rates of the selected countries. Based on our core model of only known pathogenic and high-confidence pLOF variants, Italy, Spain, and Japan are expected to have much smaller *PEX1*-ZSD populations. These estimates increase for expected intermediate and mild patients in Italy and Spain when we expand our models to include predicted pathogenic variants. This increase is driven in part by enrichment for *PEX1* variant, NC_000007.14:g.92494369C>T, NM_000466.3:c.2954G>A, p.(Arg985His), which is currently classified as a variant of uncertain significance in ClinVAR (ClinVarID: 911570). The best estimates for each country stratified by expected phenotype can also be found in Supplemental Table 7.

### Comparison of observed patient genotypes in the United States with the core equilibrium model

**Figure 5:**
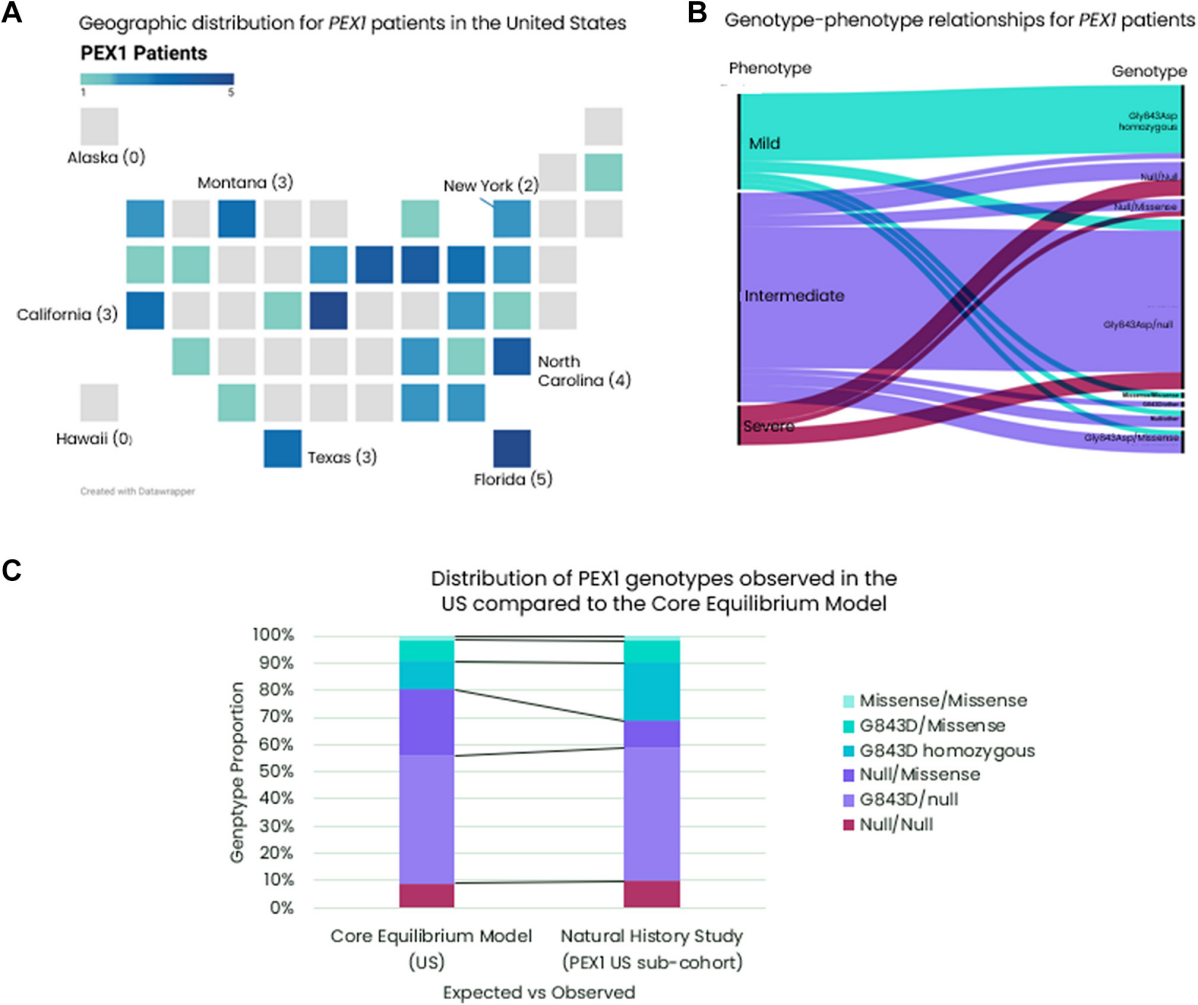
**Comparison of the core equilibrium model with observed patients from the Natural History Study for the United States subcohort.** A. Distribution of 61 patients across the United States currently enrolled in the international, Longitudinal Natural History Study (LNHS) (ClinicalTrials.gov ID: NCT01668186). States in light gray indicate no PEX1 patients enrolled. Select states are labeled for orientation. B. Distribution of Genotypes and Phenotypes observed for the PEX1 US subcohort of the LNHS Study. The leftmost column indicates the proportion of phenotypes as classified by clinical criteria described in Bose et al^10^ and flows into the corresponding genotype class. Colors indicate the clinical phenotype, with red being severe, purple representing intermediate disease severity, and teal representing mild disease severity. C. Distribution of genotypes observed in the LNHS PEX1 US subcohort compared with the expected proportion of genotypes predicted by the core equilibrium model for the United States.

To better understand the potential value of these models we compared the genotype frequencies observed in the largest patient cohort from the current Longitudinal Natural History Study of patients with Peroxisomal Biogenesis Disorder (ClinicalTrials.gov ID: NCT01668186). Specifically, we compared the subcohort of *PEX1* patients from the United States with the expected genotype frequencies based on the corresponding core model based on known pathogenic variants, reflecting current diagnostic trends. Patients in the US subcohort are distributed across the country, with more populous states contributing more patients, with the notable exception of Montana (Figure 5A). Enrichment of patients in Montana may indicate a locally higher frequency of heterozygous persons for pathogenic variants (2 of 3 were p.(Gly843Asp) homozygous, mild patients) and/or increased diagnosis rates. We also observe that patient’s genotype-phenotype relationships largely follow the assignments we applied in our models (Figure 5B). However, when comparing the observed genotype proportions with the expected number of patients with different genotypes based on the core model, we note that the observable patient population fails to meet absolute equilibrium (Χ^2^ goodness of fit, *P* = .0169). Nevertheless, the proportions expected are not excessively different than observed genotypes except for p.(Gly843Asp) homozygous genotypes (Figure 5C). Enrichment for homozygous genotypes is indicative of underlying population substructure that is not sufficiently captured in the current model.

## Discussion

Previous studies estimated the incidence of ZSD resulting from deleterious variants in any of the 13 possible *PEX* genes to be between 1 in 50,000 to 83,000 births.^1^^,^^31^ Earlier data from Japan estimated that the even broader category of peroxisomal biogenesis disorders (which includes *PEX7*-mediated disease) occurred only once in 500,000 births.^32^ By comparison, our conservative, core model suggests that ZSD specifically due to *PEX1* variants occurs ∼1 in 245,000 births in the United States and as frequently as 1 in 114,000 births based on our expanded model. We note that the data set for Japan is smaller and less representative than those applied for the United States and Europe, resulting in a less robust model, but it suggests that we expect far fewer *PEX1*-ZSD patients in Japan. Nevertheless, efforts to improve diagnosis of less severe ZSD cases are underway in many regions, including Japan, by the implementation of broader metabolomics, newborn screening initiatives, and next-generation sequencing.^33^, ^34^, ^35^, ^36^, ^37^

We have applied the same principles of inheritance as earlier attempts to model ZSD based on population genetics approaches,^31^ but we are able to overcome many of the substantial data limitations that hampered earlier calculations. Previous studies relied on the ExAC database of 60K individuals, whereas we are now able leverage over 1.2 million genomes to greatly improve representation of different geographies. Combined with the mounting clinical evidence for specific *PEX1* pathogenic variants and our improved ability to predict pathogenicity in silico with the adoption of machine learned algorithms, we could more effectively capture the genetic diversity in *PEX1* and predict the likelihood of pathogenic genotypes.

The other key advance in our model is the robust survival data^10^ and genotype-phenotype relationships that have been described in recent years, allowing us to translate birth incidence to disease prevalence. In total our best estimates for *PEX1*-ZSD based only on known variants suggests that there are approximately 500 patients, the majority exhibiting an intermediate phenotype, across the United States, United Kingdom, Germany, France, Italy, Spain, and Japan (Figure 4), with distinct genetic variant and geographic distributions. We have also compared our core equilibrium model with currently diagnosed patients in the United States, demonstrating that, although the observable patient population does not meet complete equilibrium, major trends are reflected in the core model, and we most likely underestimate p.(Gly843Asp) homozygosity.

When we extend our model to include predicted pathogenic variants, an additional ∼260 patients with intermediate phenotype are predicted across the same regions (Figure 4). Most notably, our model also indicates an additional ∼930 patients exhibiting milder features of ZSD, under the age of 31 years old (Figure 4). Our expanded model is presumably less reliable than our core model given the challenges in predicting variant pathogenicity from sequence alone; however, we also expect our expanded model to better capture the genetic diversity of this autosomal recessive disorder, as well as milder phenotypes that are less easily diagnosed. Indeed, a growing number of mild *PEX1*-ZSD patients are being diagnosed and reported with the advent of available genetic testing.^38^, ^39^, ^40^, ^41^ These patients typically present with normal cognition, retinal dystrophy, hearing loss, and enamel abnormalities and are sometimes initially misdiagnosed with Usher Syndrome. The term Heimler Syndrome, encompassing the additional features of amelogenesis imperfecta and nail dystrophy, was also used to describe this phenotype before the genetic etiology was known (and includes patients with hypomorphic alleles in *PEX1* and *PEX6*).^42^^,^^43^

Despite the advances of this model, there are numerous limitations that will drive both positive and negative errors in our epidemiologic estimates. We do assume the Hardy-Weinberg equilibrium in our populations over a broader geography that does not capture the higher likelihood to produce offspring with people in the same region, resulting in possible variant enrichment. An example of higher geographic resolution data sets to uncover pockets of increased risk for autosomal recessive disorders was recently published.^44^

In addition, we have not accounted for potential fetal mortality,^45^^,^^46^ which may overestimate severe cases. Because of sequencing approaches, we also miss large variants, including deletions and copy-number variants, as well as deep intronic variants^39^ that can also contribute to *PEX1*-ZSD. This model could be further improved with additional diverse and representative cohorts and improvements to variant calling. Furthermore, exceptions to our generalization that missense variants are associated with milder phenotypes have been reported, as well as specific pairings of missense alleles could lead to a severe effect on PEX1 protein function.^47^^,^^48^ As additional patients are diagnosed, we expect to be able to further clarify genotype-phenotype correlations, including any potential impact of genetic background

This model represents the continued evolution of our understanding of *PEX1*-ZSD and our ability to harness increasing genetic diversity data to provide key insights into genetic rare and orphan diseases. Unlike classical epidemiology approaches based on counting diagnosed patients, this model of *PEX1*-ZSD births and total disease prevalence is independent of diagnosis. Not surprisingly, our expanded model suggests a preponderance of mild patients, as well as additional intermediate patients. These estimates should provide a reliable indication to the scale of unmet need in this challenging disease and drive improvements in closing the diagnosis gap for patients with intermediate and mild ZSD.

## Data Availability

All data used to build our models are available from public sources as noted in the methods and specific variants listings are included in the Supplemental Tables.

## Conflict of Interest

The authors declare no conflicts of interest.

## References

[1] Argyriou C., D’Agostino M.D., Braverman N. (2016). Peroxisome biogenesis disorders. Transl Sci Rare Dis.

[2] Falkenberg K.D., Braverman N.E., Moser A.B. (2017). Allelic expression imbalance promoting a mutant PEX6 allele causes Zellweger spectrum disorder. Am J Hum Genet.

[3] Waterham H.R., Koster J., Ebberink M.S. (2023). Autosomal dominant Zellweger spectrum disorder caused by de novo variants in PEX14 gene. Genet Med.

[4] Rush E.T., Goodwin J.L., Braverman N.E., Rizzo W.B. (2016). Low bone mineral density is a common feature of Zellweger spectrum disorders. Mol Genet Metab.

[5] Steinberg S., Chen L., Wei L. (2004). The PEX Gene Screen: molecular diagnosis of peroxisome biogenesis disorders in the Zellweger syndrome spectrum. Mol Genet Metab.

[6] Schwerter D.P., Grimm I., Platta H.W., Erdmann R. (2017). ATP-driven processes of peroxisomal matrix protein import. Biol Chem.

[7] Berendse K., Klouwer F.C.C., Koot B.G.P. (2016). Cholic acid therapy in Zellweger spectrum disorders. J Inherit Metab Dis.

[8] Berendse K., Engelen M., Ferdinandusse S. (2016). Zellweger spectrum disorders: clinical manifestations in patients surviving into adulthood. J Inherit Metab Dis.

[9] Majewski J., Wang Z., Lopez I. (2011). A new ocular phenotype associated with an unexpected but known systemic disorder and mutation: novel use of genomic diagnostics and exome sequencing. J Med Genet.

[10] Bose M., Yergeau C., D’Souza Y. (2022). Characterization of severity in Zellweger spectrum disorder by clinical findings: a scoping review, meta-analysis and medical chart review. Cells.

[11] Lee J., Yergeau C., Kawai K., Braverman N., Géléoc G.S.G. (2022). A retrospective study of hearing loss in patients diagnosed with peroxisome biogenesis disorders in the Zellweger spectrum. Ear Hear.

[12] Yergeau C., Coussa R.G., Antaki F., Argyriou C., Koenekoop R.K., Braverman N.E. (2023). Zellweger spectrum disorder: ophthalmic findings from a new natural history study cohort and scoping literature review. Ophthalmology.

[13] Taliun D., Harris D.N., Kessler M.D. (2021). Sequencing of 53,831 diverse genomes from the Nhlbi TOPMed Program. Nature.

[14] All of Us Research Hub | Aggregated Data Browser. National Institutes of Health. https://databrowser.researchallofus.org/.

[15] Collins R.L., Brand H., Karczewski K.J. (2020). A structural variation reference for medical and population genetics. Nature.

[16] Mitsuhashi N., Toyo-oka L., Katayama T. (2022). TogoVar: a comprehensive Japanese genetic variation database. Hum Genome Var.

[17] McLaren W., Gil L., Hunt S.E. (2016). The Ensembl variant effect predictor. Genome Biol.

[18] Jaganathan K., Kyriazopoulou Panagiotopoulou S., McRae J.F. (2018). Predicting splicing from primary sequence with deep learning. Cell.

[19] Canson D.M., Davidson A.L., de La Hoya M. (2023). SpliceAI-10k calculator for the prediction of pseudoexonization, intron retention, and exon deletion. Bioinformatics.

[20] Frazer J., Notin P., Dias M. (2021). Disease variant prediction with deep generative models of evolutionary data. Nature.

[21] Cheng J., Novati G., Pan J. (2023). Accurate proteome-wide missense variant effect prediction with AlphaMissense. Science.

[22] Rosewich H., Ohlenbusch A., Gärtner J. (2005). Genetic and clinical aspects of Zellweger spectrum patients with PEX1 mutations. J Med Genet.

[23] Agresti A., Coull B.A. (1998). Approximate is better than “exact” for interval estimation of binomial proportions. Am Stat.

[24] Federal Reserve economic data Federal Reserve Bank in St. Louis. https://fred.stlouisfed.org/.

[25] People, population and community. Office for National Statistics. https://www.ons.gov.uk/peoplepopulationandcommunity.

[26] Home. Eurostat. https://ec.europa.eu/eurostat.

[27] Ministry of Health, Labour and Welfare. https://www.mhlw.go.jp/english/database/db-hw/vs01.html.

[28] Braverman N.E., Raymond G.V., Rizzo W.B. (2016). Peroxisome biogenesis disorders in the Zellweger spectrum: an overview of current diagnosis, clinical manifestations, and treatment guidelines. Mol Genet Metab.

[29] Tamura S., Matsumoto N., Imamura A. (2001). Phenotype-genotype relationships in peroxisome biogenesis disorders of PEX1-defective complementation group 1 are defined by Pex1p-Pex6p interaction. Biochem J.

[30] Alshenaifi J., Ewida N., Anazi S. (2019). The many faces of peroxisomal disorders: lessons from a large Arab cohort. Clin Genet.

[31] Vasiljevic E., Ye Z., Pavelec D.M., Darst B.F., Engelman C.D., Baker M.W. (2019). Carrier frequency estimation of Zellweger spectrum disorder using ExAC database and bioinformatics tools. Genet Med.

[32] Shimozawa N., Nagase T., Takemoto Y., Ohura T., Suzuki Y., Kondo N. (2003). Genetic heterogeneity of peroxisome biogenesis disorders among Japanese patients: evidence for a founder haplotype for the most common PEX10 gene mutation. Am J Med Genet A.

[33] Thistlethwaite L.R., Li X., Burrage L.C. (2022). Clinical diagnosis of metabolic disorders using untargeted metabolomic profiling and disease-specific networks learned from profiling data. Sci Rep.

[34] Takashima S., Saitsu H., Shimozawa N. (2019). Expanding the concept of peroxisomal diseases and efficient diagnostic system in Japan. J Hum Genet.

[35] Enns G.M., Ammous Z., Himes R.W. (2021). Diagnostic challenges and disease management in patients with a mild Zellweger spectrum disorder phenotype. Mol Genet Metab.

[36] Shimozawa N., Takashima S., Kawai H. (2021). Advanced diagnostic system and introduction of newborn screening of adrenoleukodystrophy and peroxisomal disorders in Japan. Int J Neonatal Screen.

[37] Kemper A.R., Brosco J., Comeau A.M. (2017). Newborn screening for X-linked adrenoleukodystrophy: evidence summary and advisory committee recommendation. Genet Med.

[38] Gao F.J., Hu F.Y., Xu P. (2019). Expanding the clinical and genetic spectrum of Heimler syndrome. Orphanet J Rare Dis.

[39] Muñoz-Pujol G., Alforja-Castiella S., Casaroli-Marano R. (2022). Diagnostic odyssey in an adult patient with ophthalmologic abnormalities and hearing loss: contribution of RNA-Seq to the diagnosis of a PEX1 deficiency. Int J Mol Sci.

[40] Barillari M.R., Karali M., Di Iorio V. (2020). Mild form of Zellweger Spectrum Disorders (ZSD) due to variants in PEX1: detailed clinical investigation in a 9-years-old female. Mol Genet Metab Rep.

[41] Lipiński P., Stawiński P., Rydzanicz M. (2020). Mild Zellweger syndrome due to functionally confirmed novel PEX1 variants. J Appl Genet.

[42] Ratbi I., Falkenberg K.D., Sommen M. (2015). Heimler syndrome is caused by hypomorphic mutations in the peroxisome-biogenesis genes PEX1 and PEX6. Am J Hum Genet.

[43] Heimler A., Fox J.E., Hershey J.E., Crespi P. (1991). Sensorineural hearing loss, enamel hypoplasia, and nail abnormalities in sibs. Am J Med Genet.

[44] Jackson S., Freeman R., Noronha A. (2024). Applying data science methodologies with artificial intelligence variant reinterpretation to map and estimate genetic disorder prevalence utilizing clinical data. Am J Med Genet A.

[45] Corsten-Janssen N., Bouman K., Diphoorn J.C.D. (2020). A prospective study on rapid exome sequencing as a diagnostic test for multiple congenital anomalies on fetal ultrasound. Prenat Diagn.

[46] Shamseldin H.E., AlAbdi L., Maddirevula S. (2021). Lethal variants in humans: lessons learned from a large molecular autopsy cohort. Genome Med.

[47] Havali C., Dorum S., Akbaş Y., Görükmez O., Hirfanoglu T. (2020). Two different missense mutations of PEX genes in two similar patients with severe Zellweger syndrome: an argument on the genotype-phenotype correlation. J Pediatr Endocrinol Metab.

[48] Alamatsaz M., Jalalypour F., Hashemi M.S., Shafeghati Y., Nasr-Esfahani M.H., Ghaedi K. (2021). Compound heterozygous p. Arg949Trp and p. Gly970Ala mutations deteriorated the function of PEX1p: a study on PEX1 in a patient with Zellweger syndrome. J Cell Biochem.

